# Breaching Learners’ Social Distancing through Social Media during the COVID-19 Pandemic

**DOI:** 10.3390/ijerph182111012

**Published:** 2021-10-20

**Authors:** Muhammad Zaheer Asghar, Ayesha Iqbal, Pirita Seitamaa-Hakkarainen, Elena Barbera

**Affiliations:** 1 Department of Education, University of Helsinki, 00014 Helsinki, Finland; pirita.seitamaa-hakkarainen@helsinki.fi; 2 School of Doctorate Education & ICT (E-Learning), Universitat Oberta de Catalunya, 08018 Barcelona, Spain; 3 Department of Engineering, Bedford College, Bedford MK42 9AH, UK; ayeshaiqbal_online@hotmail.com; 4 Faculty of Psychology and Education Sciences, Universitat Oberta de Catalunya, 08018 Barcelona, Spain; ebarbera@uoc.edu

**Keywords:** academic performance, blended learning, COVID-19, social media

## Abstract

Higher education has been shifted toward blended learning during the COVID-19 pandemic. An increase in social media usage intensity and reduced face-to-face interaction due to the COVID-19 pandemic urged instructional communication researchers to revisit the dynamics of learners’ group development in terms of their socialization and academic performance during the COVID-19 crisis. This research aimed to determine the mediating role of social media sociability between face-to-face socialization and academic performance of higher education students in blended learning environments during the COVID-19 pandemic. It was also the aim of the study to determine the moderating effect of social media usage intensity on social media sociability. A cross-sectional survey was conducted with the students (*n* = 340) enrolled in science teacher education departments of universities in Pakistan. Partial least squares structural equation modeling (PLS-SEM) was used for multivariate analysis. Results revealed that face-to-face socialization gave an essential start to develop a learning group. However, when face-to-face socialization was reduced due to the COVID-19 pandemic, it was mediated by social media usage in blended learning environments to increase their socialization and academic performance during the crisis. The findings of the study are useful for higher education institutions to adopt social media strategies for students’ socialization during the crisis.

## 1. Introduction

The recent global spread of the COVID-19 pandemic, the infectious disease caused by the recently discovered SARS-CoV-2 coronavirus, has severely affected the global economy, businesses, tourism, social life, and education. As COVID-19 cases and the number of deaths are gradually being controlled worldwide, most educational institutes are now considering and formalizing new rules and guidelines for the continued provision of learning while maintaining students’ safety and well-being at the same time. In the current scenario, blended learning or hybrid learning emerged as a beneficial and effective solution [1]. Blended learning uses various combinations of learning approaches, ranging from face-to-face learning (i.e., traditional classroom setups) and social media platforms (i.e., Zoom, Facebook groups, and WhatsApp groups) to online learning (i.e., using the Internet and other sources for distance and remote learning) [2]. The Higher Education Commission of Pakistan (HEC) has also recommended that the universities should begin blending, as well as the online teaching–learning process [3]. However, critics have expressed growing concerns about the quality of online instruction, faculty members’ preparedness, the nature and delivery mechanisms of exams and means of evaluation, students’ connectivity, and socialization challenges [4].

Sociability is defined as a tendency to interact with others, and instead of living alone, preferring to affiliate with others [5]. Mehall [6] said that socialization plays an essential role in the learning process. The socialization of learners is a procedure of exchanging knowledge, skills, and attitude with other learners and teachers in face-to-face and online learning environments [7]. Students’ social isolation due to social distancing in online environments became a big challenge for effective learning [8]. Online classes were not sufficient and did not enhance the learners’ socialization for an improved learning experience [9]. According to Swani et al. [10] and Wu [11], social media networks provide a high level of sociability and attractiveness to their users.

Face-to-face interaction is considered an important factor to enhance socialization in a normal situation, while social media has replaced face-to-face interaction to enhance the socialization of the learners during the COVID-19 pandemic [12,13]. Social media has also emerged as an essential platform for enhancing student interaction, sociability, and learning; and connecting faculty with students and their mentors [14]. WhatsApp, Facebook, YouTube, and Wikipedia are considered top-rated media for scholarly communication [15].

Governmental reports, social media insights, and surveys showed a trend of increase in social media usage worldwide during the pandemic as compared to before the pandemic. For example, the China Internet Information Centre [16] reported an increase in social media usage during the pandemic of 3.2 h per week as compared to prior pandemic social media usage among Chinese people [17]. The intensity of social media usage on platforms such as Facebook, Instagram, and YouTube was increased from March to April 2020, and it remained persistent throughout the COVID-19 lockdown [18,19]. A longitudinal Twitter analysis study from January to April 2020 showed a significant increase in tweets in the USA [20]. Vodafone official data [21] showed a 50% increase in Internet usage during the pandemic in India. Another survey from India showed that an increase in Internet usage was mostly due to an increase in social media usage during a crisis [22]. A survey study from Cyprus reflected a 50% increase in social media traffic in response to the COVID-19 pandemic [23]. Researchers utilized different research approaches to interpret the data of change in social media usage trends [24,25] during the crisis for different segments of society to guide decision makers in their areas of study, such as a gender studies research article [26] that interpreted the results of an increase in social media usage during a pandemic by sexual minorities and heterosexual groups for their psychological health. The study endeavored to interpret the effects of the increase in social media usage trends in the field of pedagogical sciences under the light of social learning theories to continue education without compromising social distancing by taking care of the physical and psychological health of the learners.

It was necessary to maintain social distancing to prevent COVID-19′s spread; while socialization is also equally important to maintain the teaching–learning process [27] during normal situations or any emergency. Prior studies related to social media in blended learning environments were limited to social media usage [28]. Researchers have less frequently studied the different aspects of social media, such as social media use intensity and its relationship with social media sociability for higher education students, especially during the COVID-19 pandemic. Despite the known importance of face-to-face socialization for e-learning [29] and social media involvement in a blended learning environment [30], the dynamics of instructional communication have been changed during the COVID-19 crisis. Miller et al. [31] suggested that although researchers have conducted huge amounts of research regarding instructional communication and learning group dynamics in online, social media, blended learning, and traditional face-to-face environments, revision of those studies is needed in the context of the COVID-19 crisis. In this way, the results of the empirical studies would provide best practices to continue education in crises and other difficult situations. In our case, social media emerged as an effective mode of socialization during the COVID-19 pandemic. Social media sociability has changed the dynamic of learning groups in face-to-face, online, and blended learning environments. Higher education students in Pakistan experienced mixed phases of online learning, face-to-face learning, and social media interaction during and after the COVID-19 lockdown. The relationships of face-to-face socialization, social media usage intensity, and social media sociability in blended learning environments are unknown in an emergency such as the COVID-19 pandemic, and need to be studied.

By keeping in view the research gap, this study aimed to survey students’ perception about the mediating role of social media sociability between face-to-face socialization and academic performance in blended learning environments (i.e., face-to-face traditional classrooms and online learning). It was also the aim of the study to assess the moderating effect of social media usage intensity in the relationship of face-to-face socialization and social media sociability. This study adopted a positivist research approach to survey preservice science education teachers or higher education students enrolled in science teacher education departments of universities located in Pakistan, and it utilized PLS-SEM to test the proposed hypotheses.

The research questions under discussion were:

(1) What is the effect of face-to-face socialization on students’ academic performance in blended (i.e., traditional face-to-face classroom and online) learning environments during the COVID-19 pandemic?
(2) Does social media usage intensity moderate the relationship between face-to-face socialization and social media sociability?
(3) Is there a mediating role of social media sociability between face-to-face socialization and academic performance of the higher education students in blended learning environments during the COVID-19 pandemic?

This research paper presents knowledge addition in three ways. First, it adds the mediating role of social media sociability in between face-to-face socialization and academic performance in the blended learning environment; as well as moderating the effect of social media usage intensity on social media sociability. Secondly, previously studied group development models are mostly meant for face-to-face learning environments, while this study focused on both face-to-face and social media interactions for learning group development in blended learning environments in terms of socialization and academic performance of the students. Learning group development models that we will discuss in the theoretical model section are mostly produced through a qualitative research approach. This article presents an empirical model through robust statistical analysis, and the results of the study can be generalized for higher education students. Thirdly, learning group development and its dynamics are mostly studied in normal circumstances, but we present empirical evidence in the context of the COVID-19 pandemic. Overall, this study presents valuable insights for teacher education departments, higher education institutions, and policymakers to develop strategies and policies to continue education in crises.

The remaining paper proceeds with the discussion of theoretical models for learning group development. A conceptual framework and hypotheses are developed based on theoretical models and previous studies. The research methodology section presents the research approach, population, sample, instrument development, and instrument description. The data analysis section presents the questionnaire reliability and validation, and the structural equation model measurement to assess direct and specific indirect paths. The discussion section presents the results in the light of previous studies, and the conclusion section sums up the study with practical implications.

## 2. Theoretical Models

This section presents theoretical model discussion based on face-to-face socialization, computer-mediated communication, the intervening role of social media, and learning group development. We proceeded with literature for linear group development, as well as sequential, nonsequential, and social media-based continuous learning group development, for the socialization of the learners and academic performance.

Michinov and Michinov [32] gave the opinion that the blended learning model comprises an initial face-to-face interaction, followed by asynchronous and synchronous online discussions, and ending with face-to-face interaction for performance evaluation. Potra et al. [33] said that face-to-face socialization of the students at the beginning provides an opportunity to know each other and develop a sense of belonging with a certain group. Delbiaggio et al. [34] suggested that if a face-to-face meeting is not possible, in that case social media can provide an alternative way of communication for students to develop a sense of belonging to a certain learning group, and can help to socialize students. For example, researchers found that an initial interaction or socialization using asynchronous or synchronous social media networks or computer-mediated communication had a positive impact on the subsequent collaboration of a group in a face-to-face learning setting [32,35]. The influence of initial face-to-face interaction on blended learning group collaboration is also considerable. Researchers [36,37] found that the initial face-to-face socialization of the students influenced their collaboration in blended learning environments. Some mixed results were also found by the researchers regarding initial face-to-face interactions for learning groups. A study [38] found that initial face-to-face socialization of the students had no effect or negative effect on online learning group formation. Wilson et al. [38] also found that shifting from face-to-face socialization of the learners toward social-media-based or computer-mediated socialization did not result in a substantial change in collaboration. A negative effect of face-to-face socialization was found by Salmon [39], in that face-to-face socialization may create a barrier later in online learning environments. However, it also was evident that most of the researchers found a positive effect of face-to-face socialization on learners’ socialization and task performance in blended learning environments [40].

Bales and Strodtbeck [41] and Tuckman [42] presented sequential models for learning group development that are useful in following a strategy for the introduction of face-to-face socialization initially, which may be followed by computer-mediated socialization, which would be helpful in learning group progression from stage to stage. The first stage in Tuckman’s [42] group development model is the face-to-face introduction of the group participants, which is meant to discover “who knows what” and where to fit in a learning group. This stage is also known as the group-forming stage. Tuckman and Jensen [43] described that the group-forming stage is followed by four consecutive stages: storming, norming, performing, and adjourning. Each step in this model is important. If one step is not completed, the latter also cannot be completed [32]. It is also worth mentioning that each step involves two processes: task behavior and interpersonal relationships [35]. According to Michinov and Michinov [32], these processes are similar to Bale’s [41] model of equilibrium, which describes that a group of learners is always in the process of maintaining a balance between expressive and instrumental needs. Mcgrath’s [44] concept of time, interaction, and performance (TIP) is also similar to the learners’ group process, which says that group development must be observed in terms of socialization and performance. Michinov and Michinov [32], Schellens and Valcke [45], and Torres [35] claimed that online learning generates fewer messages than face-to-face learning, but it also accommodates by compensating more performance-oriented messages. Therefore, it is significant to notice all aspects of group development, rather than considering one aspect. It also helps to understand the pattern of how groups are formed in blended learning environments between face-to-face and online learning interaction. According to Strijbos et al. [46], linear progressive models have been utilized to determine group development in online environments. Their study showed that in the beginning stages, online learning group members’ messages were focused on knowing each other and maintaining an environment of trust [47]. In the next stage, they sent comments and messages to interact with each other for task performance [48].

Models of nonsequential group development have also been introduced, which suggest that face-to-face interaction may also be more useful at other intervals of online learning rather than at the beginning point. Gerisck [49] introduced a punctuated equilibrium model to analyze the patterns of socialization and task performance behavior of a group over time. This model states that instead of developing gradually, learning groups show stability at the first level, and then show revolutionary changes and dramatic changes when a deadline is approaching. These two periods can be divided into three points: beginning, midpoint, and end. In the first stage of group formation, which begins with the first interaction of the students, the group members set a frame of action that will help them to achieve the objectives until the transition point is reached halfway through the collaborative work. When the learning group reaches its midpoint, a need for urgency to complete the task is triggered among the group members. This transition point is such a moment in which a learning group encounters a serious deadline to achieve the tasks [32,50].

Scholars agree with the group development stages of Tuckman and other models, but they do not agree with the linear and sequential application of the models [51], whether in an online or blended learning environment [52]. The communication process and group development dynamic have been catalyzed with the introduction of innovative technologies and communication sources such as social media. Social media provides continuous support as an alternative to face-to-face interaction in group development stages from the perspective of the studies mentioned above, from linear group development to nonsequential group development [53]. Group development has become a continuous process in the presence of innovative technologies. Frisby et al. [54] suggested that learning groups working together in blended learning situations should develop in the way that Tuckman’s model proposed. They appreciated both asynchronous and synchronous tools for group development to achieve every stage, with a focus on socialization and performance simultaneously.

According to Frisby et al. [40], the importance of both socialization and performance-based group development is grounded in the rhetorical and relational goal theory (RRGT) of instruction by Mottet et al. [55]. Learning groups need to set both goals of socialization and academic performance to complete the tasks and academic work [54]. According to studies by Wrench et al. [56] and Knoster et al. [57], which tested RRGT, the existence of the interdependence of relational and rhetorical sets of goals was confirmed. These instructional perspectives were similar to previous research that presented computer-mediated communication to achieve group performance, as well as socialization [35,54]. Thus, technology may help students efficiently manage both the relational and rhetorical requirements of group work. Technology plays an important role in enhancing group members’ interactions. The selection of appropriate technology is essential by keeping in view the purpose of interaction instead of choosing a conveniently available source. Information and communication technologies (ICTs) include email, instant messaging, and social media. ICTs that closely resemble face-to-face interaction are considered appropriate for socialization and task performance. The basic factors that are considered in the selection of appropriate ICTs for interaction purposes are those that enable group members to connect on the different points of view, provide effective and flexible two-way communication, ensure understanding of each other’s ideas, and convey sensitivity or emotional feelings [58]. Another challenge in the selection of ICTs for socialization is the perspective of the message senders and receivers. Closed-loop interaction, a procedure that requires the follow-up of interaction with the recipient of a message in the group to ensure the delivery and understanding of the message, can be useful [59]. In this regard, social media networks are proven more effective for understanding the feeling, socialization, and delivery of messages in a nonformal way [60].

Social media networks are online resources that are utilized for communication, creative expression, and shared learning to strengthen academic work in higher education [61]. Social networking sites and applications play an important role in knowledge sharing in education systems, especially in the social sciences [62,63]. Rasheed et al. [64] said that collaboration with peers through social media tools provides an opportunity to share knowledge with others about real-life experiences within higher education. According to Eden [65] students’ participation in social media groups influences their wellbeing, emotional development, collaborative learning, self-esteem development, and academic success. Another study by Malik et al. [66] found a significant positive effect of social media usage on the academic performance of the students, and presented that students with social media usage achieve better results as compared to nonusers. According to Mirascieva et al. [67] the use of social media network sites gave students in higher education a sense of being part of a community. Shamsudin [68] studied the effects of social media on the academic performance of Malaysian undergraduate students, and it was found that social media showed a positive and significant effect on the students’ learning and their collaboration with peers, mentors, and friends.

We conclude the theoretical discussion with researchers’ [69,70] suggestion that if a face-to-face meeting is not possible, in that case social media platforms and chat rooms or any other synchronous tools can provide an alternative way of communication for students to develop a sense of belonging to a certain learning group. It will help to socialize students [71] and develop broader opportunities for the learner in broader perspectives for collaborative learning [72]. For example, researchers have found that an initial interaction or socialization using asynchronous or synchronous social media networks or computer-mediated communication had a positive impact on the subsequent collaboration of a group in face-to-face, online, and blended learning settings [73].

### 2.1. Hypotheses Development

Our first hypothesis regards the influence of face-to-face socialization on academic performance in blended learning environments. Researchers [74,75] said that learning groups that have fewer opportunities to interact face-to-face have a high probability of encountering low-performance issues. Face-to-face interaction develops a sense of belonging to a learning group among students. Face-to-face socialization helps students to know each other and socialize. According to Brady [76], social learning theory applies efficiently in face-to-face interaction to socialize students for changes in behavior and learning. Therefore, we developed the following hypothesis:

**Hypothesis** **1.** *Face-to-face socialization has a positive influence on the academic performance of university students in blended learning environments*.

Individuals who interact face-to-face also tend to interact in the virtual environments of social media networks. Several studies are available that researched the positive effect of face-to-face socialization on interpersonal relationship development in social media networks [35]. Face-to-face socialization influences socialization in social media environments [77]. When students were confined to their homes during COVID-19, they tended to communicate with their friends, classmates, and mentors in social media environments whom they already knew through face-to-face interaction setups such as classrooms. Keeping in view the above discussion, the second hypothesis was proposed as follows:

**Hypothesis** **2.** *Face-to-face socialization positively affects socialization in social media environments among university students*.

Social media plays an important role in the socialization of online learning groups through sharing information, as well as community formation, collaboration, and extension [78]. The more social media is used effectively, especially for socialization, collaboration, and communication in online blended programs, the higher the level of student satisfaction is observed [79]. Different social media tools help in the socialization of students, and according to social learning theory, when learners interact with their classmates, teachers, and mentors, they learn from each other. Hence, the third hypothesis was developed as follows:

**Hypothesis** **3.** *Social media sociability affects the academic performance of university students in blended learning environments*.

Social media usage intensity is defined by two main aspects: the degree and frequency of social media usage, and a person’s attitude toward social media [80,81]. Previous studies have proposed that the intensity of social media usage positively influences socialization in online environments when individuals also interact with each other face-to-face [80,82]. Keeping in view the above discussion, we proposed the following fourth hypothesis:

**Hypothesis** **4.** *Social media use intensity moderates between the relation of face-to-face socialization and social media sociability*.

During the COVID-19 pandemic, students utilized social media to interact with their friends whom they knew in face-to-face settings, which helped them to reduce their feeling of social isolation, and ultimately enhanced academic performance. According to Ellison et al. [81], mostly social media users from the same organization know each other through face-to-face interaction before interacting in online environments, which helps them to enhance their job performance. It may apply to higher education students from the same institution to enhance their academic performance in blended learning environments. The intervening role of social media between face-to-face socialization and group performance is evident from previous studies [83,84]. The above-mentioned discussion led us to propose the following fifth hypothesis:

**Hypothesis** **5.** *Social media sociability plays a mediating role between face-to-face socialization and academic performance in blended learning environments*.

### 2.2. Conceptual Framework

**Figure 1:**
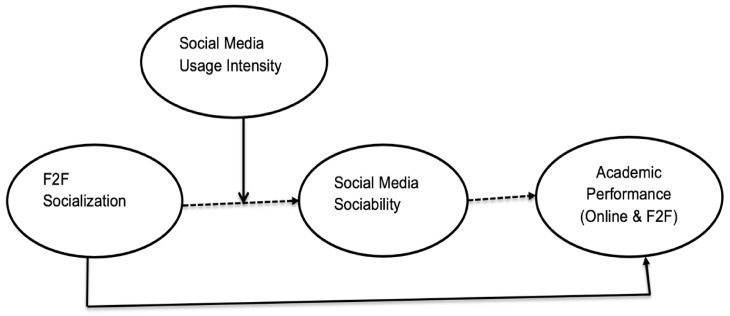
The conceptual framework.

A conceptual framework was developed in the light of the theoretical discussion. Face-to-face socialization was taken as an exogenous construct; the intensity of social media usage was taken as moderate between the relation of face-to-face socialization and social media sociability; social media sociability was considered as mediating construct between face-to-face socialization and academic performance; and academic performance was considered an endogenous construct, which led to the following hypothetical model development shown in Figure 1.

## 3. Methodology

### 3.1. Research Approach

This study used a survey-based questionnaire approach. The survey approach was adopted for three reasons. First, the influence of face-to-face socialization and the use of social media required a self-reported questionnaire to understand the academic performance of higher education students in blended learning environments. Secondly, it required a sufficient number of respondents to generalize the results of the study to the population. Thirdly, higher education students were easily accessible in our contacts to complete the questionnaires.

### 3.2. Questionnaire Development

The questionnaire comprised three major parts. The first part contained the title, introduction, ethical statement, and respondent consent to participate in the study as a volunteer. The second part comprised demographics of the respondents, such as gender, age, and qualification. The third part comprised three main constructs of face-to-face socialization: social media usage intensity, social media sociability, and academic performance through online and face-to-face approaches. Initially, the questionnaire comprised 37 items on a Likert-type scale, ranging from 1 = strongly disagree to 5 = strongly agree. The instrument reliability and validity were measured through pilot testing. The questionnaire was distributed among 20 Ph.D. students and 10 researchers. The Cronbach’s alpha value of the reliability of the questionnaire was found to be above 0.8, which was satisfactory. Respondents also provided their feedback regarding the content validity of the questionnaire. The items of the questionnaire were revised according to the feedback of the respondents.

Exploratory factor analysis (EFA) was used for data reduction, which reduced the total number of items to 29. The reliability of the questionnaire was found satisfactory (Cronbach’s alpha = 0.943). EFA was applied with the extraction method of principal component analysis (PCA). Varimax with Kaiser normalization was used as a rotation method. The sample was found adequate, with Kaiser–Meyer–Olkin (KMO) = 0.924, and Bartlett’s test of sphericity was significant (*p* < 0.001).

### 3.3. Construct Measurements

We provided the detailed constructs along with items in Supplementary Material S1. The final questionnaire comprised the following constructs.

#### 3.3.1. Face-to-Face Socialization (FS)

This construct comprised six items adapted from previous studies [85,86,87,88]. A sample of three items of the construct is given as follows: “I do not feel lonely in life”; “I am able to make spontaneous informal face-to-face conversations with others”; and “I am able to do non-task-related face-to-face conversations with others”. A construct was considered reliable with a Cronbach’s alpha above the threshold of 0.7 [89]. The construct was consistent and reliable, with a Cronbach’s alpha value = 0.801.

#### 3.3.2. Social Media Sociability (SS)

This construct comprised six items adapted from previous studies [85,86,87,88]; a sample of three selected items of the construct is given as follows: “Social media environment enables me to easily contact to my teammates”; “I do not feel lonely in this social media environment”; and “Social media environment enables me to get a good impression of my teammates”. The construct was consistent and reliable, with a Cronbach’s alpha value = 0.763.

#### 3.3.3. Social Media Usage Intensity (SI)

This construct comprised five items adapted from previous studies [78,81]. A sample of four selected items is given as follows: “Social media is part of my everyday activity”; “Social media has become a daily part of my routine”; “I feel out of touch when I have not logged on to social media for a while”; and “I am proud to tell people I’m on social media”. The construct was consistent and reliable, with a Cronbach’s alpha value = 0.853.

#### 3.3.4. Online Academic Performance (OP)

This construct comprised seven items adapted from previous studies [85,90]. A sample of three selected items is given as follows: “Online learning environment enables me to develop my academic skills with other best students”; “I know what I want in academics and online learning environment facilitates me to get it”; and “Through online environment, friends encourage and collaborate with me in my academics more”. The construct was consistent and reliable, with a Cronbach’s alpha value = 0.834.

#### 3.3.5. Face-to-Face Academic Performance (FP)

This construct comprised five items adapted from previous studies [85,90]. A sample of three selected items is given as follows: “I am able to develop my academic skills with other best students in face-to-face classes”; “I enjoy meeting new people face-to-face who would enrich me in my subject knowledge”; and “I can talk about my career goals with other people face-to-face when I require it”. The construct was consistent and reliable, with a Cronbach’s alpha value = 0.811.

### 3.4. Sample and Data Collection

Preservice science education teachers or students in science education programs enrolled in the education departments of universities located in Pakistan were the target population of the study. The institutional ethical committee reviewed the proposal for ethical considerations. The participants of the study completed the consent form before filling out the survey. A G*Power calculator was used to compute the sample size required for this research, which used a structural equation model (SEM), and anticipated a required minimum sample size = 350, given the model’s structural complexity [91,92].

Higher education in Pakistan has seen mixed phases of teaching–learning approaches during the pandemic. At the beginning of the COVID-19 pandemic, a strict lockdown was imposed, due to which mobility of teachers and students was restricted to their houses. The higher education commission of Pakistan issued a notification to shift the higher education institutions to online learning. Students were again shifted to the traditional learning setup when the severity of the pandemic was reduced after a few months in Pakistan. The face-to-face learning process could not continue due to the forecast of the second wave of the COVID-19 pandemic. The government of Pakistan introduced the idea of a smart lockdown to prevent the spread of the pandemic. The smart lockdown was meant to close vicinities that crossed a certain level of pandemic spread. Higher education institutions were advised to utilize blended learning approaches by keeping in view the need for smart lockdowns in their vicinity. The data were collected from the students who studied through face-to-face approaches before the COVID-19 pandemic breakout, then shifted to online learning during the COVID-19 lockdown, and finally they joined classes through blended learning approaches during the smart lockdown.

The online platform Qualtrics.com was used to collect the data from respondents. The questionnaire was distributed among 400 students of the teacher education departments that offered a degree program in science education in Pakistan. A total number of 340 surveys was finalized after data screening. The response rate was 85%.

### 3.5. Demographics

The sample distribution of the 340 respondents showed that males (52%) and females (48%) were almost equally represented in the study; the majority (68%) of them were between the ages of 18 to 21, and fewer respondents (7%) were 26 years old or above; the majority (60%) of the respondents were BS students, while master’s students (37%) also responded, and there was also a minor representation (0.02%) of Ph.D. students. The distribution of the sample is presented in Table 1.

## 4. Data Analysis

This research used Smart_PLS 3.2.8 software for data analysis. Structural equation modeling based on partial least squares (SEM-PLS) is considered the best technique for multivariate analysis, as it helps in theory development [89]. A two-step analysis procedure was adopted in this research, as suggested by researchers [93]. The first step was performed to evaluate the outer model; construct reliability and divergent and convergent validity was measured. The second step was performed to evaluate the inner model; effect size (*f*^2^), coefficient of determination (R^2^), the goodness of fit model, VIF values, and PLS predict were measured.

### 4.1. Measurement Model Evaluation

The outer model was evaluated through measuring constructs’ reliability, observed variables’ validity in relation to unobserved constructs, and validity of single observed constructs. Single observed variables may be retained with an outer loading minimum of 0.4, while 0.7 is considered satisfactory to include in relevant constructs [94]. The outer loading of the items ranged from 0.668 to 0.866, as given in Table 2. The composite reliability (CR) was found adequately greater than 0.7 for all factors [95]. The average variance extracted was also found adequate for all factors (AVE > 0.5). According to [96,97], reliability values of the Cronbach’s alpha, rho alpha, and composite reliability (CR) must be greater than 0.7.

The reliability values of the Cronbach’s alpha, rho alpha, and composite reliability (CR) for individual factors were above the threshold of 0.7, as shown in Table 2.

The heterotrait–monotrait ratio of correlations (HTMT) was used to assess the divergent validity of the constructs [98,99]. Henseler et al. [100] presented HTMT to assess the constructs’ discriminant validity. According to Sarstedt et al. [101] an HTMT value below the threshold of 0.9 is considered satisfactory. The HTMT values of all constructs were below the threshold of 0.9, as given in Table 3.

#### Second-Order Factor Analysis

A second-order factor analysis was performed that showed that face-to-face academic performance (W = 0.352, *p* < 0.0001) and online academic performance (W = 0.75, *p* < 0.0001) were significant formative subconstructs of the academic performance, as given in Table 4.

The descriptive analysis showed that social media use (M = 3.33, SD = 0.94), academic performance (M = 3.41, SD = 0.84), and face-to-face socialization (M = 3.46, SD = 0.80) tended toward agreement, as shown in Table 5.

### 4.2. Structural Model Measurement

The outer model was found appropriate, with reliable and valid constructs. The inner model was measured in the next step, which comprised the measurement of R^2^, predictive relevance, and the goodness of fit model.

#### 4.2.1. Coefficient of Determination (R^2^)

R^2^ measures a model’s predictivity, which represents the explained variance and its influence on the structural model. The academic performance endogenous construct showed an R^2^ = 0.588, and endogenous construct social media use showed an R^2^ = 0.409. It was suggested that R^2^ values must be above the threshold of 0.10 [93,102]. All the R^2^ values were above the threshold level of 10%, as given in Table 6.

#### 4.2.2. Predictive Relevance

**Figure 2:**
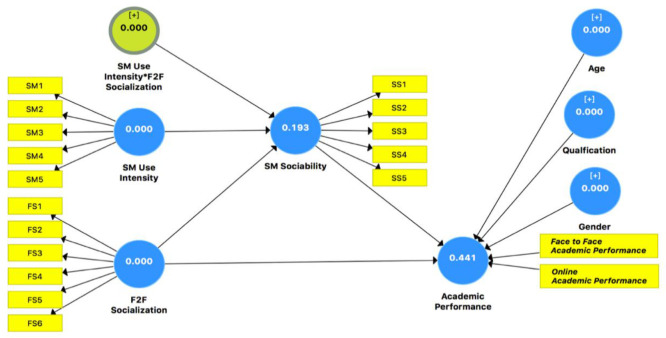
The redundancy analysis.

Geiser [103] introduced the Stone–Geisser’s Q^2^ value to assess the criterion predictive accuracy along with the measurement of R^2^ values. The path model quality was measured with cross-validity redundancy analysis to determine the Stone–Geisser’s Q^2^ value through a blindfolding procedure. It showed the effectiveness of the model to predict endogenous constructs. SEM should show a value of Q^2^ above the threshold of zero for endogenous constructs. The Q^2^ (= 1 − SSE/SSO) values were observed at above the threshold of zero for all endogenous constructs, as shown in Figure 2. This showed that SEM was appropriate for the predictive relevance of endogenous constructs.

#### 4.2.3. Goodness of Fit

Tenenhaus et al. [104] suggested that goodness of fit (GOF) is useful to explain the empirical data for the effectiveness of a model. The path model is considered valid for GOF values above 0.36 (substantial) and 0.25 (moderate), and below 0.10 as weak. The plausibility and parsimoniousness of the model are measured through GOF values. The GoF formula is GoF = sqrt ((average AVE) × (average R^2^)). The GoF value was found to be 0.495, which showed a satisfactory level of the model’s validity, as shown in Table 7.

The standardized residual (SRMR) represents the index between hypothesis covariance matrices and observed values [105]. The SEM estimated fit is measured through SRMR. Hu and Bentler [106] said that a path model with SRMR < 0.08 is considered a good fit. THE SRMR values of the SEM in this study were observed to be less than the threshold of 0.08, and the NFI value was above the threshold of 0.8. Another measure that was considered for the goodness of fit was the VIF value. VIF measures the collinearity issues of the constructs. According to Hair et al. [97], the VIF value must be below a threshold of 5 to avoid collinearity issues. The inner model VIF values were observed to be below the threshold of 5, which meant there was no collinearity issue, as shown in Table 8.

#### 4.2.4. Structural Model Path Analysis

The mean values of the path coefficient were considered the same as β values in the regression path coefficient. According to the methods of Grimsey and Lewis [107], the hypotheses were tested with β values. The per-unit variation independent construct due to independent construct was represented by β. Significance values, as well as t-statistics, were used to verify β values. According to Chin [108], the bootstrapping procedure is useful to measure significance level. The direct path coefficients, t-stats, and significance values at a bootstrapping level of 5000 are given in Table 9.

Demographic variables such as gender, qualification, and age showed no effect on endogenous construct, with *p* > 0.05. Face-to-face socialization showed an influence on academic performance (β = 0.473, *t* = 10.313, *p* < 0.0001). Hence, Hypothesis 1 was accepted. Face-to-face socialization showed a positive and significant influence on social media use (β = 0.373, *t* = 7.149, *p* < 0.0001). Hence, Hypothesis 2 test was accepted. Social media use showed a positive and significant influence on academic performance (β = 0.396, *t* = 8.198, *p* < 0.0001). Hence, Hypothesis 3 was accepted, as shown in Table 9.

#### 4.2.5. Moderating Effects

Social media usage intensity showed a direct positive and significant effect on social media sociability (β = 0.360, *t* = 7.004, *p* < 0.0001). This led us to test the interaction of the effect of social media usage intensity with face-to-face socialization on social media sociability, which was nonsignificant (*p* > 0.05). Hence, Hypothesis 4 was not accepted, as shown in Table 10.

#### 4.2.6. Specific Indirect Effect

The indirect specific path showed the significant and positive mediating effect of social media between face-to-face socialization and academic performance (β = 0.148, *t* = 5.855, *p* < 0.0001). Hence, Hypothesis 4 was accepted, as shown in Table 11.

**Figure 3:**
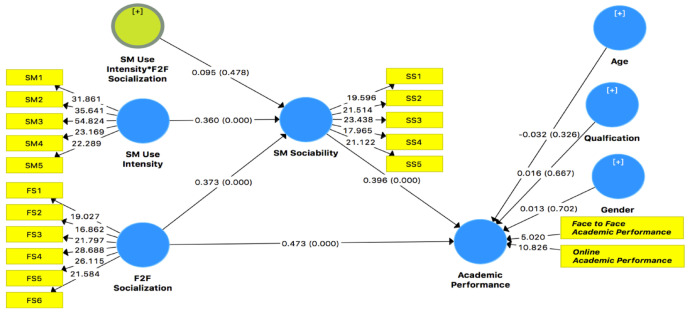
The path analysis.

The overall direct paths are shown in Figure 3.

## 5. Discussion

It was the aim of the study to determine the influence of face-to-face socialization on social media sociability and academic performance during the COVID-19 pandemic. The moderating role of social media usage intensity concerning face-to-face socialization and social media sociability was also studied. It was also the aim of the study to assess the mediating role of social media sociability between face-to-face socialization and academic performance through blended learning approaches during the COVID-19 pandemic.

The current study showed that preservice science teachers perceived that face-to-face socialization had a positive influence on their academic performance in blended learning environments. Hence, Hypothesis 1 was accepted. Previous studies also highlighted the relationship between F2F socialization and academic performance [109]. Hammond [110] showed that there existed a significant relationship between academic performance and F2F social integration. The population of this study was based on students enrolled in teacher education programs in science education. It was important for them to attend labs and perform practical work. They could not perform practical work without face-to-face interaction. Social interaction of preservice science education helped them through social learning, which resulted in face-to-face socialization being a key component for science students to enhance their academic performance.

The findings of the current study revealed that face-to-face socialization influenced the social media sociability of preservice science teachers. Hence, Hypothesis 2 was accepted. The result of the study coincided with previous research, which found that when certain people interact to form a group, social media provides a substitute for socialization in virtual spaces for face-to-face socialization [111]. Students actively socialized themselves in social media environments with their friends whom they knew face-to-face before the COVID-19 pandemic [112,113]. Social media provided an alternative to socialization for higher education students whose friends, teachers, and mentors were present in a social media environment.

This study noticed a direct and positive effect of social media usage intensity on social media sociability. This led us to determine the interaction effect of social media usage intensity and face-to-face socialization on social media sociability. It was a knowledge addition of the study that social media usage intensity was linked to enhancing the sociability of students in crisis. However, the moderating effect of social media usage intensity between the relationship of face-to-face sociability and social media sociability was not observed. Hence, Hypothesis 4 was rejected. Previous research endorsed the relationship between social networks and sociability in a normal situation [80,114]. Researchers [12,115,116] also presented the effects of social media usage on users’ sociability in social media networks. Hence, social media usage intensity was directly linked with the sociability of the students during a crisis such as the COVID-19 pandemic. However, the effect of interaction in terms of social media usage intensity and face-to-face socialization was not observed, because social distancing minimized the face-to-face interaction during the COVID-19 pandemic.

It was also a finding of this study that social media sociability influenced students’ academic performance in blended learning environments. Hence, Hypothesis 4 was accepted. Kassens-Noor [117] found out that social software such as Facebook, blogs, wikis, and Twitter are frequently embedded in instructional designs to support learning nowadays, especially during the COVID-19 pandemic [118]. Social media tools enhance academic performance, whether online or offline, such as peer assessment, content creation and sharing, collaborations, individual and group learning experiences, and upgrading information to help perform learning tasks. Shiu et al. [119] found that Facebook was an excellent supplementary source to provide an alternative to classroom socialization. Twitter is considered a useful social media platform for information sharing, making connections, and seeking social assistance from others [120]. Preservice science teachers’ socialization via social media helps them to share ideas and knowledge, which motivates them to perform actively in online and face-to-face learning environments.

The current study found that social media sociability intervened between face-to-face socialization and academic performance in blended learning environments. Hence, Hypothesis 5 was approved. The finding of the study coincided with previous research [81,83]. Mayrberger and Linke [121] found that social networks could positively change a student’s participation rate in online and face-to-face learning environments. Whittle and Bickerdike [122] found out that first-year science students’ orientation helped them to socialize on social media networks, which ultimately helped them to benefit from practical work. Preservice science teachers met their classmates before the pandemic face-to-face, which helped them to socialize in social media environments, and which ultimately enhanced their academic performance when blended learning was introduced during the COVID-19 pandemic.

This research also found that students’ demographics, such as age and gender, had no impact on their academic performance, whether it was online or face-to-face. A study by Coldwell et al. [123] found out that academic performance did not show a significant relationship between age and other demographics.

## 6. Conclusions

This study was conducted to find the influence of face-to-face socialization and social media sociability on learning group dynamics in blended learning environments during the COVID-19 pandemic. It was concluded that learners’ face-to-face socialization played a significant role in enhancing the academic performance of the students in blended learning environments during the crisis. The face-to-face interaction of higher education students, especially in science education, cannot be ignored, even in a crisis. Hence, it is also important to take care of social distancing in the pandemic. Therefore, face-to-face socialization can be induced in social-media-based socialization. Social media usage was linked with the socialization of students during the crisis, which showed an influence on enhancing the academic performance of the students. When blended learning approaches were introduced during the COVID-19 pandemic, higher education students’ socialization on social media not only enhanced their academic performance in face-to-face learning environments, but also helped to increase their academic performance in hybrid online environments. Thus, it was concluded that face-to-face socialization provided an initial kickstart for blended learning groups to know each other and understand the nature of the learning process. Social media usage was a source of continuous socialization when face-to-face interaction of the higher education students is not possible. Social media usage helped students to understand the social learning environments and perform academic tasks as humans, instead of learning robotically. Social media sociability as a mediator between face-to-face socialization and academic performance guaranteed the student’s protection from pandemics by maintaining social distancing and learning success by providing them a socialized environment for social learning.

### Implications

Education should never be stopped in an emergency, whether it is a war, pandemic, or natural disaster, because this education will prepare people to take them out of crises in the long run. However, the value of human lives is also significant in protecting learners from emergencies’ side effects. Maintaining social distance was essential to developing an environment of socialization for the teaching–learning process. Health experts recommended social distancing during the COVID-19 crisis, which meant eliminating face-to-face learning.

- A blend of different learning approaches should be utilized in a pandemic, ranging from face-to-face interactions and social media to online learning.
- Science education students must attend laboratories to perform practical work. Science education institutions may devise strategies to allow students’ visits to laboratories in a restricted way for limited numbers of students by taking care of social distancing.
- Teachers may devise social media groups to enhance the interaction of students. The students’ face-to-face interactions in laboratories would also help to enhance students’ participation in social media groups to improve the teaching–learning experience.
- Devising such social media strategies that students utilize more, and more institutional-based social media networks that include student’s classmates, mentors, and teachers, are needed. The face-to-face interaction and social media groups would ultimately enhance academic performance in practical work at laboratories and theoretical discussion in online learning environments.
- Future studies may be conducted based on this research to find an appropriate blend of the learning approaches for in-service science teachers’ professional development during an emergency.

## Figures and Tables

**Table 1:** Distribution of the sample characteristics.

Characteristics	f	%
Gender		
Male	176	52
Female	164	48
Age		
18 to 21	229	68
22 to 25	89	25
26 and above	22	7
Qualification		
BS	205	60
Master’s	126	37
Ph.D.	9	0.02

**Table 2:** Constructs’ validity and reliability.

Construct	Item	Loading	œ	rho_A	CR	(AVE)
Face-to-face academic performance	FP1	0.753	0.811	0.812	0.864	0.515
	FP2	0.738				
	FP3	0.729				
	FP4	0.705				
	FP5	0.690				
Face-to-face socialization	FS1	0.757	0.801	0.804	0.858	0.502
	FS2	0.749				
	FS3	0.707				
	FS4	0.684				
	FS5	0.682				
	FS6	0.667				
Social media intensity	SI1	0.866	0.853	0.861	0.895	0.631
	SI2	0.806				
	SI3	0.805				
	SI4	0.751				
	SI5	0.738				
Online academic performance	OP1	0.739	0.834	0.836	0.876	0.501
	OP2	0.731				
	OP3	0.726				
	OP4	0.718				
	OP5	0.692				
	OP6	0.681				
	OP7	0.668				
Social media sociability	SS1	0.741	0.763	0.763	0.840	0.513
	SS2	0.723				
	SS3	0.718				
	SS4	0.7				
	SS5	0.699				
	SS6	0.741				

**Table 3:** Heterotrait–monotrait ratio of correlations (HTMT).

	FP	FS	SI	OP
F2F academic performance				
F2F socialization	0.724			
Social media usage intensity	0.435	0.525		
Online academic performance	0.724	0.834	0.627	
Social media sociability	0.679	0.73	0.712	0.827

**Table 4:** Second-order factor analysis.

	Weight	Sig.
Face-to-Face Academic Performance > Academic Performance	0.352	0.000
Online Academic Performance > Academic Performance	0.752	0.000

**Table 5:** Descriptive statistics.

Constructs	M	SD
Social media sociability	3.33	0.94
Academic performance	3.41	0.80
Face-to-face socialization	3.46	0.80
Social media intensity	3.32	0.93

**Table 6:** R^2^ coefficients of determination.

	R^2^	R^2^ Adjusted
Academic performance	0.588	0.582
SM sociability	0.409	0.404

**Table 7:** The Goodness of fit index.

Constructs	Average Variance Extracted (AVE)	R^2^
Face-to-face academic performance	0.515	0.588
Face-to-face socialization	0.502	0.409
Social media intensity	0.631	
Online academic performance	0.501	
Social media sociability	0.513	
Average values	0.532	0.498
GOF	0.495	

**Table 8:** The VIF and model’s goodness of fit.

Constructs	VIF		Goodness of Fit Model
	Academic Performance	Social Media Sociability	
F2F socialization	1.488	1	SRMR = 0.057 NFI = 0.888
Social media sociability	1.49		

**Table 9:** The direct paths.

Hypothesis	*β*	*t*-Value	*p*-Value
Age → Academic Performance	−0.032	0.972	0.331
Gender → Academic Performance	0.013	0.375	0.708
Qualification →Academic Performance	0.016	0.434	0.665
F2F Socialization → Academic Performance	0.473	10.313	0.000
F2F Socialization → SM Sociability	0.373	7.149	0.000
SM Sociability → Academic Performance	0.396	8.198	0.000

**Table 10:** The moderating effect.

Hypothesis	*β*	*t-*Stats	*p*	Status
SM Use Intensity → SM Sociability	0.360	7.004	0.000	Rejected
SM Use intensity × F2F Socialization → SM Sociability	0.096	0.719	0.472	

SM = social media; F2F = face-to-face.

**Table 11:** Specific indirect path.

Hypothesis	*β*	*t*-Stats	*p*	Status
F2F Socialization → SM Sociability → Academic Performance	0.148	5.855	0.000	Accepted

## Data Availability

The data presented in this study are openly available in FigShare at https://doi.org/10.6084/m9.figshare.16592609.v1 (accessed on 18 October 2021).

## Supplementary Materials

The following are available online at https://www.mdpi.com/article/10.3390/ijerph182111012/s1, File S1: Questionnaire’s Constructs and Items.
