# Supplementary material for: Breaching Learners’ Social Distancing through Social Media during the COVID-19 Pandemic
Source: Int J Environ Res Public Health. 2021 Oct 20;18(21):11012. doi: 10.3390/ijerph182111012 (PMC8583489; doi:10.3390/ijerph182111012)
Supplement: Supplementary file 1 [file ijerph-18-11012-s001.zip › ijerph-1412646-supplementary.pdf]

(Supplementary File)
Questionnaire's Constructs and Items

Face-to-face socialization

1- I do not feel lonely in life.
2- I am able to make spontaneous informal face-to-face conversations with others.
3- I am able to do non-task-related face-to-face conversations with others.
4- I am able to easily contact face-to-face my classmates or significant others.
5- I feel comfortable while face-to-face talking with class mates and others easily
6- I am able to easily contact face-to-face my classmates or significant others.

Social media sociability

7- Social media environment enables me to easily contact to my teammates.
8- I do not feel lonely in this social media environment.
9- Social media environment enables me to get a good impression of my teammates.
10- Social media environment allows for non-task-related conversations
11- Social media environment allows spontaneous informal conversations.

Social media usage intensity

12- Social media is part of my everyday activity.
13- Social media has become a daily part of my routine.
14- I feel out of touch when I have not logged on to social media for a while.I am proud to tell people I'm on social media.
15- I would be sorry if social media shut down
16- I feel I am part of the social media community

Online academic performance

17- Online learning environment enables me to develop my academic skills with other best students.
18- I enjoy meeting new people in online classroom who would enrich me in my subject knowledge.
19- I know what I want in academics and online learning environment facilitates me to get it.
20- I can talk about my career goals with other people in online classroom when I require it.
21- Through online environment, friends encourage and collaborate with me in my academics more.
22- Online learning environment enabled me to develop my communication skills to interact with others for learning
23- Online learning environment provided me opportunities to collaborate with my classmates for extra curricular activities

Face-to-face academic performance

24- I am able to develop my academic skills with other best students in face-to-face classes.
25- I enjoy meeting new people face-to-face who would enrich me in my subject knowledge.
26- I can talk about my career goals with other people face-to-face when I require it.
27- My friends encourage and collaborate with me in my academics more in a face-to-face classroom setting.
28- I know what I want in academics and face-to-face learning environment facilitates me to get it.
